# Initial Viral Inoculum Determines Kinapse-and Synapse-Like T Cell Motility in Reactive Lymph Nodes

**DOI:** 10.3389/fimmu.2019.02086

**Published:** 2019-09-06

**Authors:** Sujana Sivapatham, Xenia Ficht, Juliana Barreto de Albuquerque, Nicolas Page, Doron Merkler, Jens V. Stein

**Affiliations:** ^1^Department of Oncology, Microbiology, and Immunology, University of Fribourg, Fribourg, Switzerland; ^2^Theodor Kocher Institute, University of Bern, Bern, Switzerland; ^3^Division of Clinical Pathology, Department of Pathology and Immunology, University Hospital of Geneva, Geneva, Switzerland

**Keywords:** T cell activation, immunological synapse, intravital imaging, viral infection, T cell motility

## Abstract

T cell activation in lymphoid tissue occurs through interactions with cognate peptide-major histocompatibility complex (pMHC)-presenting dendritic cells (DCs). Intravital imaging studies using *ex vivo* peptide-pulsed DCs have uncovered that cognate pMHC levels imprint a wide range of dynamic contacts between these two cell types. T cell—DC interactions vary between transient, “kinapse-like” contacts at low to moderate pMHC levels to immediate “synapse-like” arrest at DCs displaying high pMHC levels. To date, it remains unclear whether this pattern is recapitulated when the immune system faces a replicative agent, such as a virus, at low and high inoculum. Here, we locally administered low and high inoculum of lymphocytic choriomeningitis virus (LCMV) in mice to follow activation parameters of Ag-specific CD4^+^ and CD8^+^ T cells in draining lymph nodes (LNs) during the first 72 h post infection. We correlated these data with kinapse- and synapse-like motility patterns of Ag-specific T cells obtained by intravital imaging of draining LNs. Our data show that initial viral inoculum controls immediate synapse-like T cell arrest vs. continuous kinapse-like motility. This remains the case when the viral inoculum and thus the inflammatory microenvironment in draining LNs remains identical but cognate pMHC levels vary. Our data imply that the Ag-processing capacity of draining LNs is equipped to rapidly present high levels of cognate pMHC when antigenic material is abundant. Our findings further suggest that widespread T cell arrest during the first 72 h of an antimicrobial immune responses is not required to trigger proliferation. In sum, T cells adapt their scanning behavior according to available antigen levels during viral infections, with dynamic changes in motility occurring before detectable expression of early activation markers.

## Introduction

Naive T cells scan membranes of Ag-presenting cells (APCs), mostly dendritic cells (DCs), for the presence of cognate peptide-loaded major histocompatibility complexes (pMHC) in lymph nodes (LNs) and other secondary lymphoid organs. How they accomplish their search has been well-studied in mouse LNs using intravital two-photon microscopy (2PM). This approach has revealed that T cells move with high speeds of 12–15 μm/min on a 3D scaffold formed by fibroblastic reticular cells inside the T cell zone, onto which DCs are attached (1–3). Dynamic motility parameters change during inflammation, as T cell speeds and directionality decline owing to cognate interactions with activated DCs. To dissect the influence of pMHC levels on T cell motility patterns, *ex vivo* activated DCs were pulsed with defined levels of cognate peptide prior to injection into recipient mice, while T cell dwell times were controlled by a short homing window. This approach identified a multistep model of T cell activation, according to which T cells dynamically respond to pMHC levels (4–8). When intermediate levels of cognate pMHC are presented on activated DCs, motile T cells scan DCs for a period of a few h (phase 1; 0–8 h post LN entry). Importantly, these transient interactions, termed kinapses, between cognate pMHC-presenting DCs and motile T cells suffice for biochemical signal integration mediated by Ca-flux, nuclear NFAT translocation, c-fos phosphorylation, and CD69 upregulation (9–12). When signals accumulate above a threshold, T cells arrest for long-term interactions with individual DCs (phase 2; 8–20 h). During this period, T cells presumably form an immunological synapse as observed in *in vitro* studies (13). After ~20 h, activated T cells detach and resume motility before starting cell division (phase 3) (14, 15). Subsequent studies have refined the 3-phase concept by showing that pulsing DCs with high amounts of peptide induces immediate arrest, i.e., instantaneous phase 2 induction (5, 7, 8). In contrast, T cells may skip phase 2, i.e., stable interactions, with DCs at very low antigen dose, yet still expand during the effector phase (6). It is now widely recognized that changes in dynamic T cell motility parameters closely correlate with interactions with APCs at specific time points, and T cell speeds have been used as surrogate marker to define kinapses and synapses *in vivo* without visualizing DCs (16).

In contrast to *ex vivo* peptide-pulsed DC, the impact of cognate pMHC levels on dynamic T cell behavior during antimicrobial immune responses has thus far not been systematically explored. It remains unclear whether the endogenous Ag-presenting capacity suffices to induce abrupt T cell arrest on DCs within < 24 h p.i. as observed with *ex vivo* peptide-pulsed DCs, where the need for Ag processing is bypassed. In addition, *ex vivo* pulsing is often performed with saturating peptide doses leading to occupancy of virtually all available MHC on DC surfaces, whereas physiological infections may only lead to a fraction of pMHC pulsed with the same cognate peptide. In such a scenario, pMHC-dependent immediate arrest of recent T cell immigrants into reactive LNs may not occur during microbial infections. On the other hand, virus infections are characterized by rapid replication and generation of high antigen levels, potentially leading to high pMHC levels. Conceivably, this may result in similar antigen presentation dynamics in draining LNs irrespective of initial viral inoculum. In addition, the initial viral load may imprint distinct interaction dynamics between T cells and DCs through Ag level-unrelated changes in the inflammatory microenvironment, e.g., through altered cytokine secretion.

Here, we addressed how varying initial inoculum of lymphocytic choriomeningitis virus (LCMV) imprinted activation marker expression in Ag-specific CD4^+^ and CD8^+^ T cells in draining LNs. We correlated these data with T cell motility patterns using 2PM of reactive LNs, defining “synapse-like” behavior as indirect correlate for prolonged contacts with APCs. Our findings uncover that the antigen-presenting machinery is readily capable of rapid and prolonged processing of a wide range of pathogen-derived proteins for presentation on MHC. Furthermore, we find that the initial viral inoculum imprints dynamic T cell behavior during microbial infections through generation of cognate pMHC, rather than other factors. Finally, our data suggest that subtle alterations of Ag-specific T cell migration parameters precede detectable expression of early activation markers, making changes in motility the most sensitive early sign of a burgeoning T cell response.

## Results

### CD4^+^ and CD8^+^ T Cell Activation and Proliferation Kinetics Correlate With LCMV Inoculum

**Figure 1:**
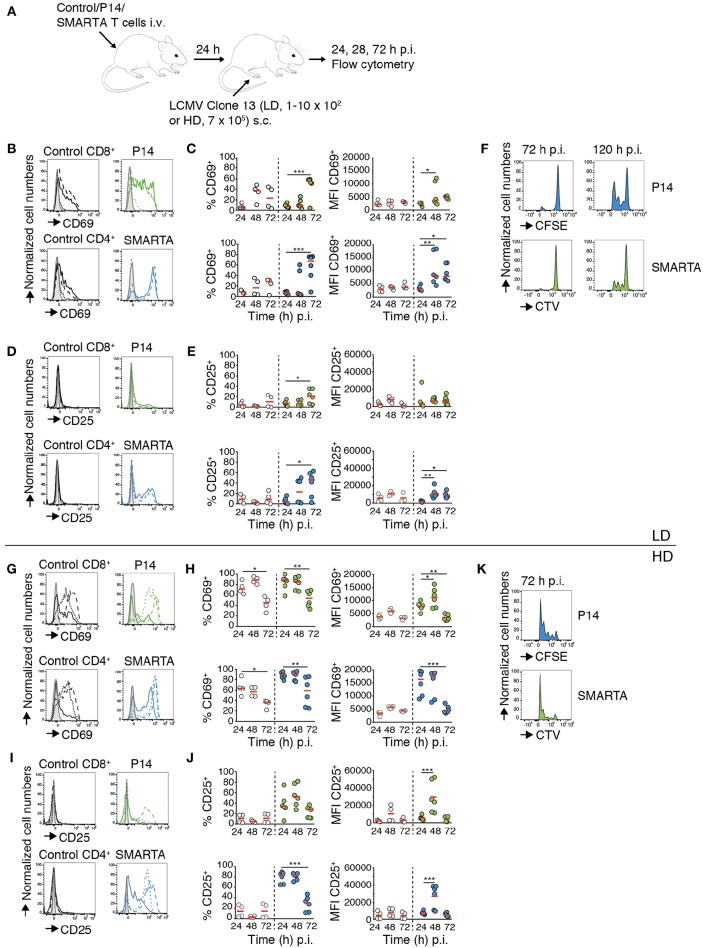
CD4^+^ and CD8^+^ T cell activation and proliferation kinetics correlate with LCMV inoculum. **(A)** Experimental layout. Activation of control T cells, P14, and SMARTA T cells was analyzed by flow cytometry at 24, 48, and 72 h post LD and HD LCMV infection. **(B)** CD69 expression on control (black), P14 (green), and SMARTA T cells (blue) at 24 (dotted line), 48 (dashed line), and 72 h (continuous line) post LD LCMV infection. **(C)** Percentage and MFI of CD69^**+**^ control (gray) and P14 (green) or SMARTA T cells (blue) post LD LCMV infection. **(D)** CD25 expression on control (black), P14 (green), and SMARTA T cells (blue) at 24 (dotted line), 48 (dashed line), and 72 h (continuous line) post LD LCMV infection. **(E)** Percentage and MFI of CD25^**+**^ control (gray) and P14 (green) or SMARTA T cells (blue) post LD LCMV infection. **(F)** Flow cytometry histograms of P14 (green) and SMARTA T cells (blue) at 72 and 120 h post LD LCMV infection. **(G)** CD69 expression on control (black), P14 (green), and SMARTA T cells (blue) at 24 (dotted line), 48 (dashed line), and 72 h (continuous line) post HD LCMV infection. **(H)** Percentage and MFI of CD69^**+**^ control (gray) and P14 (green) or SMARTA T cells (blue) post HD LCMV infection. **(I)** CD25 expression on control (black), P14 (green), and SMARTA T cells (blue) at 24 (dotted line), 48 (dashed line), and 72 h (continuous line) post HD LCMV infection. **(J)** Percentage and MFI of CD25^**+**^ control (gray) and P14 (green) or SMARTA T cells (blue) post HD LCMV infection. **(K)** Flow cytometry histograms of P14 (green) and SMARTA T cells (blue) at 72 h post HD LCMV infection. Each dot represents data from one mouse. Red lines depict median. Data are pooled from three independent experiments with a total of four to six mice per condition and time point. Statistical significance was analyzed using one-way ANOVA test. ^*^p < 0.05; ^**^p < 0.01; ^***^p < 0.001.

To examine the impact of virus inoculum on T cell responses, we transferred fluorescently labeled LCMV gp33–41 peptide-specific P14 TCR tg CD8^+^ T cells (“P14”) and LCMV gp61-80 peptide-specific SMARTA TCR tg CD4^+^ T cells (“SMARTA”) together with a polyclonal T cell population (“Control”) into C57BL/6 mice. One day post transfer, we infected recipient mice with a low (1–10 × 10^2^ pfu; low dose, LD) or high (7 × 10^5^ pfu; high dose, HD) inoculum of LCMV clone 13 via s.c. foot hock injection. At 24, 48, and 72 h p.i., we isolated footpad-draining popliteal and non-draining brachial LNs for flow cytometry (Figure 1A). Our analysis of draining LNs after LD LCMV infection showed a significant increase in both percentage of CD69^+^ and level of CD69 expression (MFI) in P14 and SMARTA T cells starting at 48 h p.i. (Figure 1B). At 72 h p.i., 55–67% of Ag-specific T cells displayed detectable surface levels of CD69 (Figure 1C). Similarly, CD25 surface markers gradually increased at 72 h after LD LCMV infection in P14 and, more pronounced, in SMARTA cells, whereas control CD4^+^ and CD8^+^ T cells did not increase surface CD25 levels (Figures 1D,E). At 72 h p.i., no proliferation of transferred P14 or SMARTA had occurred. In turn, 3–4 generations of proliferated T cells were detected at 120 h post LD infection (Figure 1F). Thus, moderate expression of activation markers in the first 72 h p.i. after LD LCMV infection correlated with T cell proliferation in the following 48 h.

We examined activation marker expression and proliferation in the HD LCMV infection model. We observed a rapid increase in percentage of CD69^+^ P14 and SMARTA T cells (88 and 89%, respectively) as early as 24 h p.i., with MFI values peaking between 24 and 48 h before decreasing at 72 h p.i. (Figures 1G,H). Control CD4^+^ and CD8^+^ T cells also increased CD69 expression on the cell surface but to lower levels as compared to Ag-specific P14 and SMARTA T cells (Figures 1G,H). CD25 expression rapidly increased in P14 and, more pronounced, SMARTA T cells as soon as 24 h p.i. and peaked at 48 h p.i. (52 and 83% CD25^+^ P14 and SMARTA T cells, respectively; Figures 1I,J). In contrast, CD25 expression remained undetectable in control T cells (Figures 1I,J). In line with these data, both P14 and SMARTA T cells had undergone effective proliferation at 72 h post HD LCMV infection (Figure 1K). In contrast, we did not detect a major increase in activation markers on P14 and SMARTA T cells in non-draining brachial LNs, suggesting that our infection model reflects a local immune response in draining LNs (Figure S1). We further examined co-stimulatory marker expression on MHC-II^+^ CD11c^+^ DCs in LD and HD-draining LNs at 24 h p.i. CD80 and in particular CD86 levels were significantly increased in HD-draining LNs as compared to non-draining or LD-draining LNs, in line with an inoculum-dependent local inflammatory response (Figure S2). In sum, both LD and HD LCMV infection triggered activation of transferred P14 and SMARTA T cells, yet with distinct kinetics and magnitude of activation marker expression and proliferation. Ag-specific T cells in LD-draining LNs displayed a delayed upregulation of activation marker expression with lower peak levels as compared to T cells of HD-draining LNs.

### Dynamic T Cell Behavior in Reactive LNs During the First 72 h of LD LCMV Infection

We set out to correlate the activation and proliferation dynamics with dynamic properties of Ag-specific T cells during LD LCMV infection. To benchmark T cell motility parameters in the absence of inflammation, we transferred P14, SMARTA, and control T cells into non-inflamed recipient mice and performed 2PM of steady-state LNs. From image sequences, we determined average track speed, meandering index (MI; actual migrated distance divided by euclidian distance between start and end point of track), arrest coefficient (percentage of track segments with speeds below 4 μm/min) and percentage of “synapse-like” motility. As surrogate readout for synapse-like motility, we defined an average track speed of <4 μm/min as threshold. This value corresponds to previously published low speeds observed during stable T cell—DC interactions in reactive LNs (7, 8). The definition of a threshold speed to determine synapse-like motility as surrogate readout for T cell interactions with APCs follows a protocol described by Bousso et al. (16). It is important to keep in mind that in our experimental system, we did not visualize APCs. Therefore, synapse-like motility does not necessarily correlate with actual interactions with APCs.

**Figure 2:**
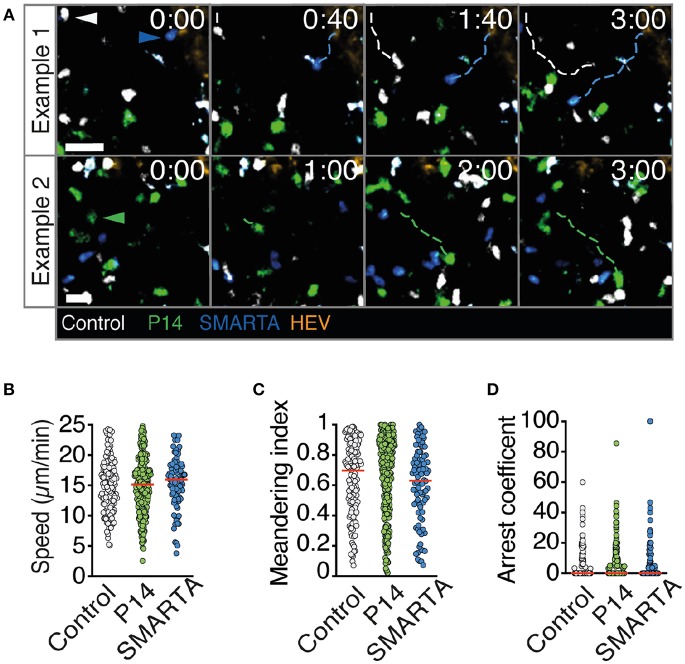
T cell motility parameters in non-infected LNs. **(A)** Representative 2PM images of control (white), P14 (green), and SMARTA T cell tracks (blue) with HEV (orange) at 96 h after adoptive T cell transfer. Arrowheads point the tracked cell: control (white), P14 (green), and SMARTA (blue). Scale bar, 20 μm. **(B–D)** Quantification of the 2PM motility parameters: Migration speed **(B)**, meandering index **(C)**, and arrest coefficient **(D)** of control (gray), P14 (green), and SMARTA T cells (blue). Red lines depict median. Each dot represents a single track. Data are pooled from two independent experiments with a total of three mice. Statistical significance in **(B–D)** was analyzed by unpaired Mann-Whitney test of control vs. P14 and SMARTA T cells, respectively.

2PM image sequence analysis uncovered high interstitial motility of P14, SMARTA, and control T cells with comparable average speeds of 15–16 μm/min (Figures 2A,B). MI values were also comparable (mean 0.60–0.66) and correlated with low arrest coefficients and complete absence of synapse-like behavior (Figures 2C,D and Video S1). Thus, all three transferred cell populations displayed an active scanning behavior in resting, non-inflamed LNs characteristic of naïve T cells during homeostasis.

**Figure 3:**
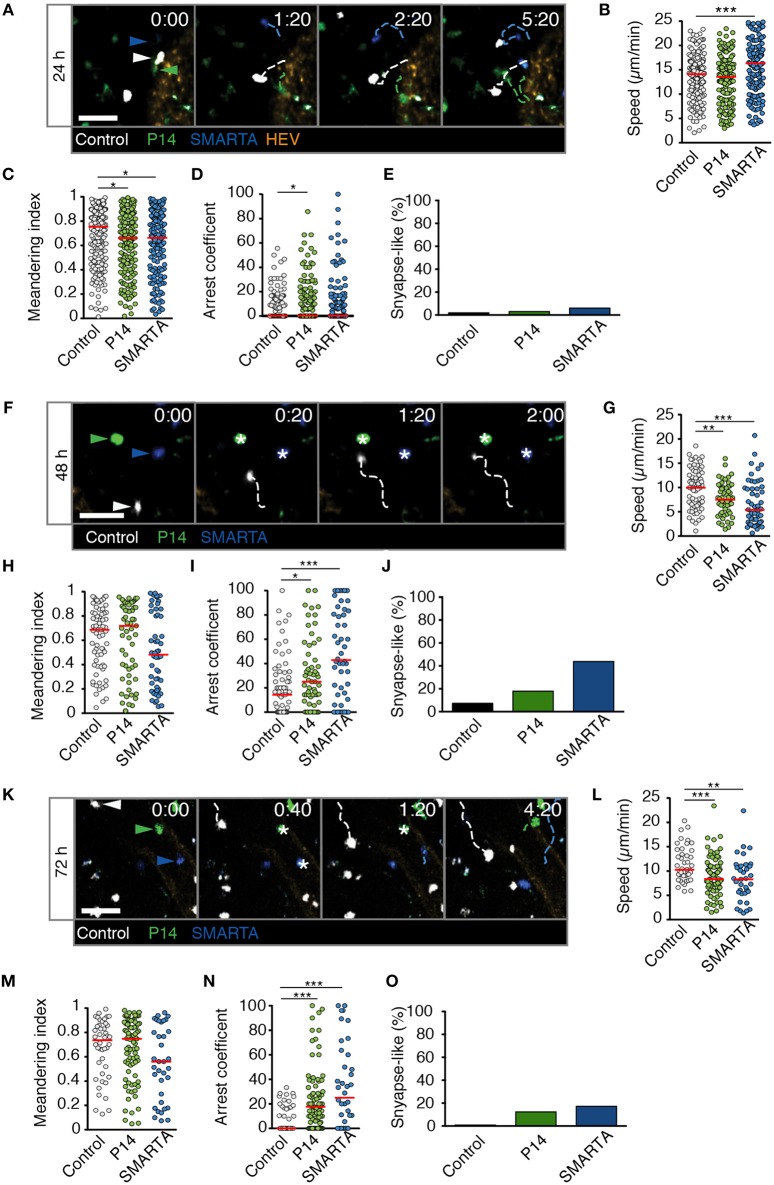
T cell motility in reactive LNs during LD LCMV infection. **(A)** Representative 2PM images of control (white), P14 (green), and SMARTA T cell tracks (blue) with HEV (orange) at 24 h p.i. Arrowheads point the tracked cell: control (white), P14 (green), and SMARTA (blue). Scale bar, 20 μm. **(B–E)** Quantification of 2PM motility parameters at 24 h p.i.: Migration speed **(B)**, Meandering index **(C)**, arrest coefficient **(D)**, and percentage of synapse-like T cell motility **(E)** of control (gray), P14 (green), and SMARTA T cells (blue). **(F)** Representative 2PM images of control, P14, and SMARTA T cells with HEV (orange) at 48 h p.i. as in **(A)**. Arrowheads point the tracked cell and * indicate arrested T cells. Scale bar, 20 μm. **(G–J)** Quantification of 2PM motility parameters at 48 h p.i.: Migration speed **(G)**, Meandering index **(H)**, arrest coefficient **(I)**, and percentage of synapse-like T cell motility **(J)** of control (gray), P14 (green), and SMARTA T cells (blue). **(K)** Representative 2PM images of control, P14, and SMARTA T cells with HEV at 72 h p.i. as in **(A)**. Arrowheads point the tracked cell and * depict arrested T cells. Scale bar, 20 μm. **(L–O)** Quantification of 2PM motility parameters at 72 h p.i.: Migration speed **(L)**, Meandering index **(M)**, arrest coefficient **(N)**, and percentage of synapse-like T cell motility **(O)** of control (gray), P14 (green), and SMARTA T cells (blue). Red lines depict median. Each dot represents a single track. Data are pooled from three independent experiments with a total of four mice (24 h), four mice (48 h), and two mice (72 h). Statistical significance was analyzed by unpaired Mann-Whitney test of control vs. P14 and SMARTA T cells, respectively. *p < 0.05; **p < 0.01; ***p < 0.001.

In LD LCMV-draining LNs at 24 p.i., all three cell populations continued to migrate with high speeds, with SMARTA T cells even displaying an increase in average cells speeds to 17.5 ± 9.7 μm/min (Figures 3A,B and Video S2). In contrast, P14 and SMARTA T cells displayed a minor but significant decrease in MI as compared to control T cells (Figure 3C). In P14 T cells, this correlated with a moderately higher arrest coefficient (Figure 3D) and low but detectable levels of synapse-like behavior in P14 and SMARTA populations at 24 h post LD LCMV infection (Figure 3E).

At 48 h post LD LCMV infection, 2PM imaging of reactive LNs revealed a decrease in P14 and SMARTA T cell speeds to 7.6 ± 3.2 and 6.7 ± 4.6 μm/min, respectively. Of note, control T cells also displayed a decrease in speeds (9.7 ± 4.1 μm/min), which was less pronounced than that of Ag-specific T cells (Figures 3F,G and Video S3). While MI values showed a non-significant decrease in SMARTA T cells as compared to control T cells (Figure 3H), decreased P14 and SMARTA speeds were reflected in higher arrest coefficients (median 14, 25, and 43% for control, P14, and SMARTA T cells, respectively) and synapse-like arrest, in particular for SMARTA T cells (44%; Figures 3I,J).

At 72 h post LD LCMV infection, control, P14, and SMARTA T cells began to recover cell speeds (11.4 ± 3.6, 8.9 ± 3.8 and 8.3 ± 4.5 μm/min, respectively), with no significant changes in MI values but increased arrest coefficient in Ag-specific T cells (0, 18, and 25% for control, P14, and SMARTA T cells, respectively; Figures 3K–N and Video S4). As a result, synapse-like motility remained detectable in P14 and SMARTA T cells, albeit at lower levels than at 48 h p.i. (Figure 3O).

In sum, our 2PM analysis detected minor alterations in Ag-specific T cell motility parameters in the first 72 h post LD LCMV infection. We did not observe widespread synapse-like behavior under these conditions, indicating that a low inoculum of a replicative agent does not necessarily lead to general T cell arrest within the first 72 h p.i. In the time windows analyzed, most Ag-specific T cells maintained robust motility throughout the observation period, suggesting “kinapse-like” transient interactions as dominant mode of T cell—DC communication. Nonetheless, it is equally possible that synapse-like behavior remains a prerequisite for full T cell activation in this setting, with the low frequency of apparent synapse formation reflecting the infrequent presence of APC.

### Dynamic T Cell Behavior During the First 72 h of HD LCMV Infection

**Figure 4:**
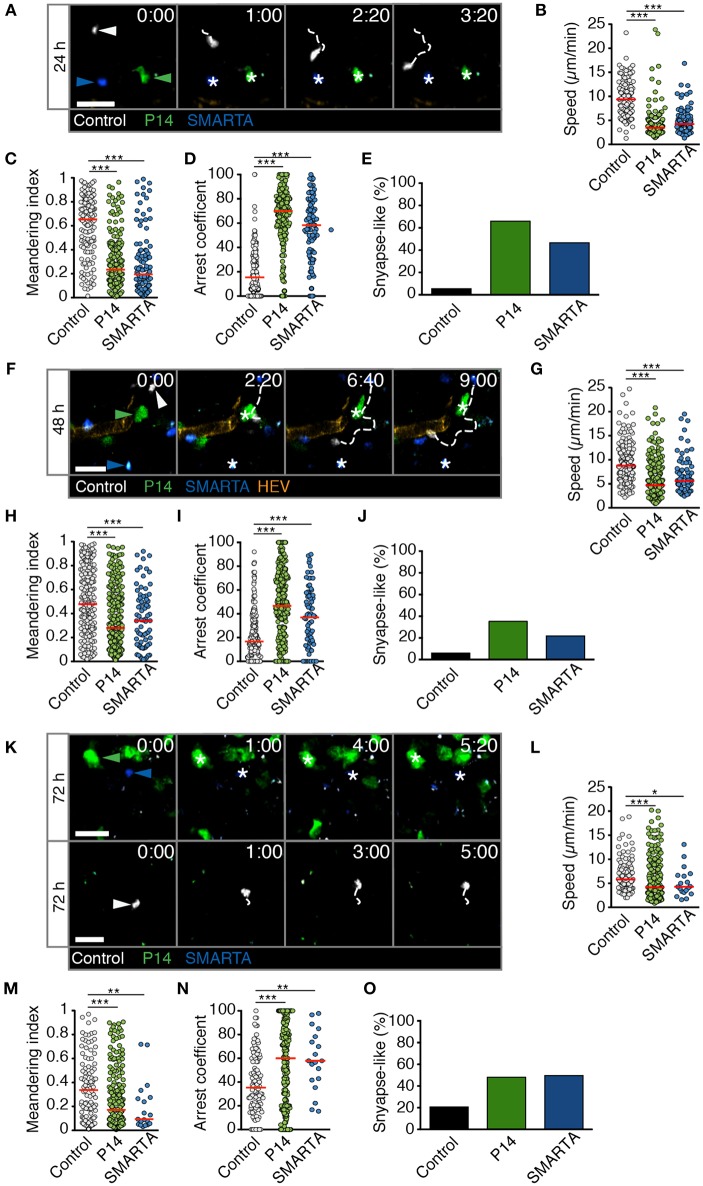
T cell motility in reactive LNs during HD LCMV infection. **(A)** Representative 2PM images of control (white), P14 (green), and SMARTA T cell tracks (blue) with HEV (orange) at 24 h p.i. Arrowheads point the tracked cell: control (white), P14 (green), and SMARTA (blue). Scale bar, 20 μm. **(B–E)** Quantification of 2PM motility parameters at 24 h p.i.: Migration speed **(B)**, Meandering index **(C)**, arrest coefficient **(D)**, and percentage of synapse-like T cell motility **(E)** of control (gray), P14 (green), and SMARTA T cells (blue). **(F)** Representative 2PM images of control, P14, and SMARTA T cells with HEV (orange) at 48 h p.i. as in **(A)**. Arrowheads point the tracked cell and * indicate arrested T cells. Scale bar, 20 μm. **(G–J)** Quantification of 2PM motility parameters at 48 h p.i.: Migration speed **(G)**, Meandering index **(H)**, arrest coefficient **(I)**, and percentage of synapse-like T cell motility **(J)** of control (gray), P14 (green), and SMARTA T cells (blue). **(K)** Representative 2PM images of control, P14, and SMARTA T cells with HEV at 72 h p.i. as in **(A)**. Arrowheads point the tracked cell and * depict arrested T cells. Scale bar, 20 μm. **(L–O)** Quantification of 2PM motility parameters at 72 h p.i.: Migration speed **(L)**, Meandering index **(M)**, arrest coefficient **(N)**, and percentage of synapse-like T cell motility **(O)** of control (gray), P14 (green), and SMARTA T cells (blue). Red lines depict median. Each dot represents a single track. Data are pooled from five independent experiments with four mice (24 h), six mice (48 h), and eight mice (72 h). Statistical significance was analyzed by unpaired Mann-Whitney test of control vs. P14 and SMARTA T cells, respectively. *p < 0.05; **p < 0.01; ***p < 0.001.

We performed analogous experiments using 2PM of reactive LNs after HD LCMV infection. At 24 h p.i., P14 and SMARTA T cell speeds and MI values had strongly decreased as compared to non-infected or LD LCMV-infected LNs (4.1 ± 2.6 and 4.8 ± 2.5 μm/min for speeds and 0.29 ± 0.23 and 0.29 ± 0.26 for MI, respectively; Figures 4A–C and Video S5). Control T cells also displayed a significant reduction in their average speeds, although less pronounced than in Ag-specific T cells (Figure 4B). Reduced P14 and SMARTA motility was mirrored in high arrest coefficients and a high percentage of synapse-like behavior (66 and 47%, respectively; Figures 4D,E). Thus, HD LCMV infection precipitates extensive arrest of Ag-specific T cells in reactive LNs within 24 h p.i.

At 48 h post HD LCMV infection, P14 and SMARTA T cells continued to display a mostly stationary behavior with low speeds (5.8 ± 3.5 and 6.7 ± 3.8 μm/min, respectively), low MI, and high arrest coefficients (Figures 4F–I and Video S6). This resulted in an elevated percentage of synapse-like motility in P14 and SMARTA T cells (36 and 22%, respectively), which was nonetheless lower than at 24 h p.i. (Figure 4J). Similar to 24 h p.i., control T cells were moving faster and more directional than Ag-specific T cells, albeit with lower motility parameters as compared to uninfected LNs (Figures 4G–I).

At 72 h post HD LCMV infection, reactive LNs had massively expanded (not shown). In 2PM image sequences, we observed continued low motility parameters in Ag-specific P14 and SMARTA T cells, with P14 T cells occasionally found in clusters (Figure 4K and Video S7). This behavior correlated with low P14 and SMARTA T cell speeds (5.8 ± 4.3 and 5.1 ± 3.0 μm/min, respectively), low MI values, increased arrest coefficients and elevated percentages of synapse-like behavior (48 and 50%, respectively; Figures 4L–O). At this time point, control T cells also displayed significantly lower speeds (6.8 ± 3.4 μm/min) and MI values (0.36 ± 0.26) as compared to 24 and 48 h post HD LCMV infection. Concomitantly, we observed increased arrest coefficients and synapse-like motility in these cells, although at lesser extent than that of Ag-specific T cells (Figures 4N,O). This behavior may reflect Ag-specific activation of a subset of polyclonal T cells. Alternatively, since our relative imaging depth as compared to the LN diameter was reduced in HD LCMV-infected LNs owing to their massive expansion, the observed control cells located in the outermost LN area, where T cells speeds are generally lower (17). Taken together, our data show that Ag-specific T cells undergo a rapid switch in their scanning behavior early during HD LCMV infections, with sustained arrest of T cell populations observed within the first 72 h p.i. inside reactive LNs.

### High Viral Inoculum Induces Immediate T Cell Arrest Through Cognate pMHC Levels in Reactive LNs

**Figure 5:**
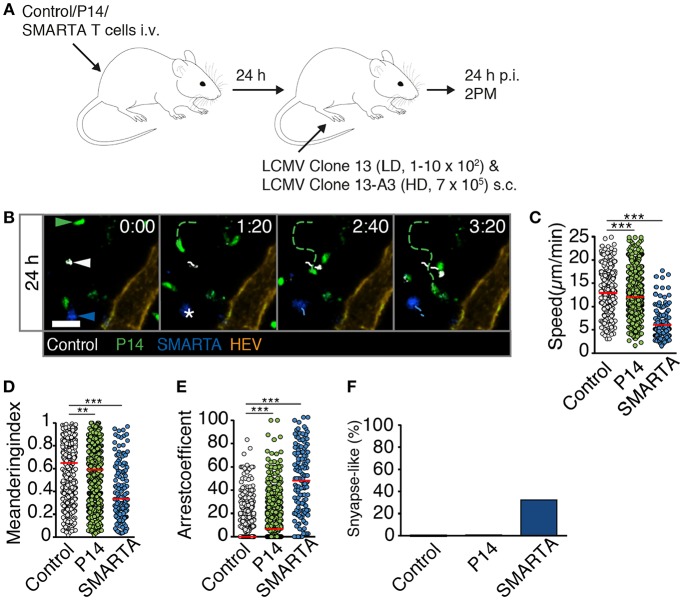
High viral inoculum induces immediate T cell arrest through high pMHC levels in reactive LNs. **(A)** Experimental layout. Fluorescently labeled control, P14, and SMARTA T cells were analyzed by 2PM microscopy at 24 h post co-infection with HD LCMV Clone 13-A3 and LD Clone 13. **(B)** Representative 2PM images of control (white), P14 (green), and SMARTA T cell tracks (blue) with HEV (orange). Arrowheads point the tracked cell and * indicates complete T cell arrest. Scale bar, 20 μm. **(C–F)** Quantification of 2PM motility parameters: Migration speed **(C)**, Meandering index **(D)**, arrest coefficient **(E)**, and percentage of synapse-like T cell motility **(F)** of control (gray), P14 (green), and SMARTA T cells (blue). Red bars depict median. Each dot represents a single track. Data are pooled from four independent experiments with a total of eight mice. Statistical significance was analyzed by unpaired Mann-Whitney test of control vs. P14 and SMARTA T cells, respectively. **p < 0.01; ***p < 0.001.

We explored the mechanisms responsible for early T cell arrest under HD infection conditions. It is conceivable that in addition to cognate pMHC, a highly inflammatory environment suffices to trigger T cell arrest even when cognate pMHC levels are low or moderate, as observed in control T cells at late time points of HD infection. This may occur via increased activation levels of APCs reflected by increased levels of co-stimulatory molecules and cytokine secretion, recruitment of circulating T cells, downregulation of promigratory factors, rapid activation marker expression or changes in intrinsic T cell motility (18–20). To explore this aspect in more detail, we transferred control, P14 and SMARTA T cells into recipient mice. One day later, we co-infected recipients with LD LCMV Clone 13 and HD LCMV Clone 13-A3. The latter virus contains a point mutation in the LCMV gp33-43 epitope preventing P14 T cell activation but preserving the SMARTA-specific epitope (21, 22). In this setting, the total viral inoculum corresponds to HD infection but cognate pMHC levels for P14 T cells correspond to LD infection. At 24 h p.i., we performed 2PM imaging of reactive LNs (Figures 5A,B and Video S8). Similar to HD LCMV infection, SMARTA T cells displayed strongly reduced speeds (6.1 ± 3.4 μm/min), low MI values and increased arrest coefficients (Figures 5C–E). This resulted in an increased synapse-like behavior as compared to control cells, reminiscent of HD LCMV infections (33%) (Figure 5F). In contrast, motility parameters of P14 T cells were remarkably similar to LD LCMV infection only. Thus, P14 T cells displayed only a minor decrease in cell speeds (12.4 ± 4.5 μm/min) and MI (0.58 ± 0.25) as compared to control T cells (13.0 ± 4.2 μm/min and 0.61 ± 0.24, respectively; Figures 5C,D). As a result, the arrest coefficient remained comparably low, with essentially no synapse-like motility pattern (Figures 5E,F). This observation suggests that cognate pMHC levels are a major determinant of early T cell arrest, even in a highly inflamed environment.

## Discussion

The aim of this study was to correlate activation and dynamic behavior of Ag-specific T cells in reactive LNs during the first 72 h p.i. with low and high viral inoculum. Our data support following conclusions: first, T cell deceleration occurs as a function of initial viral load, similar to results obtained with transferred DCs pulsed with varying peptide concentrations or potency (5–9). Second, the innate immune system is capable to process abundant virus material for presentation on MHC at levels sufficient to induce T cell arrest within 1 day post infection. Under high initial viral inoculum, cognate pMHC may be presented in high levels on the surface of APC, and/or distributed on an increased number of APCs. Third, even very low doses of initial virus inoculum induce a noticeable alteration in Ag-specific T cell motility, which precedes detectable surface expression of early activation markers such as CD69 and CD25.

One concern with previous observations using *ex vivo* peptide-pulsed DCs was that this approach created pMHC levels not attainable during physiological viral infections, since it bypasses Ag processing and can lead to uniform cognate peptide presentation on all available MHC molecules. Our data show that at least with the experimental system used here, pMHC-dependent T cell arrest can be observed within 1 day after viral infection. Our reductionist experimental system bears several limitations: the inoculum used may not represent levels of initial viral load under physiological conditions. In addition, we neither determined the levels of cognate pMHC on individual APCs nor their frequency. It remains therefore unknown whether T cell arrest is a consequence of their interactions with increased numbers of APCs presenting viral antigen and/or whether individual APC present high levels of cognate pMHC. Furthermore, the transfer of high numbers of Ag-specific T cells required for 2PM experiments may impact viral infection kinetics or competition for access to pMHC-presenting DCs. It is therefore likely that the presence of supraphysiologic numbers of P14 and SMARTA T cells will dominate over the endogenous response, as reflected in low activation of endogenous T cells shown in Figure 1. Starting 3–4 days after HD viral infection, transferred effector T cells will conceivably contribute to efficient virus elimination, thus altering the dynamic of a natural virus infection. Effector T cell egress may take longer under LD conditions, as cell proliferation in reactive LNs becomes apparent only at 5 days p.i. (Figure 1). While we have not checked the functionality of SMARTA and P14 effector T cells under LD infection, systemic infection of as few as 2 × 10^2^ pfu LCMV-WE leads to robust effector T cell generation (23).

In the LD model used here, the percentage of CD69^+^ and CD25^+^ Ag-specific T cells continues to increase up to 72 h p.i., while the MFI of both activation markers tends to peak at 48 h p.i. Similarly, 2PM data showed that Ag-specific T cells speeds recover by 72 h post LD infection as compared to 48 h p.i., while synapse-like behavior declined at this time point. Thus, our data suggest that the dynamic behavior of Ag-specific T cells is fundamentally distinct under LD and HD conditions throughout the activation process, as suggested by studies using distinct pMHC loading (4–8). Nonetheless, we cannot exclude that priming dynamics may simply be shifted in the LD infection model toward later time points, with long-term synapse-like interactions occurring after 72 h p.i., the latest time point we have examined.

During natural infections, the frequency and activation status of antigen-specific T cells varies between pathogens and changes with age, which likely impacts on the dynamic nature of T cell—DC interactions (24). Nonetheless, our observations suggest that the innate immune system is capable to react to very high antigen doses for protein processing and MHC loading. Comparable results were reported by other groups, who have observed rapid T cell arrest (within < 24 h p.i.) in reactive LNs in other virus models (25–27). Similarly, in sterile immunization models, T cells decelerate within <1 day (1, 9, 28–30). Taken together, pMHC processing appears highly efficient over a wide range of initial starting material.

Our data further support the concept that cognate pMHC levels are not the only determinant for T cell motility, since the overall level of inflammation had an impact on control T cell migration. One potential factor responsible for the overall decline in control T cell motility is thromboxane A2 released by activated DCs, and which induces a meandering behavior in naive T cells through Rho signaling (31, 32). Furthermore, strong inflammation precipitates a loss of CCL21 and CCL19 expression, factors which maintain high basal motility in naive T cells (18, 19). Yet, within the 24 to 48 h p.i. time window, pMHC levels appear as a decisive factor for T cell deceleration. This became particularly evident in the mixed infection model, where high cognate pMHC levels caused a loss of motility of antigen-specific T cells (SMARTA) without affecting non-reactive T cells (P14) despite a highly proinflammatory environment.

Here, we have not visualized DCs nor addressed the precise phenotype of APCs. In pilot 2PM experiments using CD11c-YFP recipients labeling macrophages and DCs (33), we found that CD11c-YFP signal became low to non-detectable after HD viral infections. This prevented us from directly assessing whether T cells were in close proximity to APCs when they showed low motility. In tissue sections, we could nonetheless identify occasional CD11c-YFP^+^ cells in close association with transferred P14 and SMARTA T cells (not shown). Furthermore, flow cytometry analysis of endogenous MHC-II^+^ CD11c^+^ APCs confirmed increased expression of CD80 and CD86 at 24 h after HD infection (Figure S2), suggesting abundant availability of activated DCs for long-term interactions with Ag-specific T cells. Nonetheless, since we could not directly visualize DCs in our system, we do not have direct evidence whether synapse-like behavior of Ag-specific T cells directly correlates with APC interactions. Yet, since control T cells remained consistently more motile than antigen-specific CD4^+^ and CD8^+^ T cells under all conditions tested, it is likely that Ag-specific T cell deceleration is at least in part driven by pMHC-driven APC interactions. This is further supported by the observation that P14 T cells remain motile in LNs draining high levels of LCMV clone 13-A3, despite the fact that costimulatory molecules on DCs increase under these conditions. These data are in agreement with findings by Bousso et al. on T cell deceleration after cognate peptide administration into recipient mice (16). It is likely that viral particles are directly transported via lymph from the site of injection to draining LNs, where they are taken up by subcapsular sinus macrophages (34). Of note, we did not attempt to determine kinetics of viral titers in reactive LNs over the first 72 h p.i. for a correlation with dynamic T cell behavior. We speculated that the relation between viral titer and viral material available for MHC presentation may not necessarily match. Thus, under HD conditions, viable and damaged viral particles may appear rapidly in afferent lymphatics, whereas LD infection may require several rounds of replication in the periphery before enough antigenic material is transported actively or passively to draining LNs. At later time points, migratory DCs may replace LN resident DCs as most prominent APC. We did not specifically examine the microenvironmental positioning of Ag-specific CD4^+^ vs. CD8^+^ T cells, which has been reported to differ depending on the infection model used (25, 26). At 72 h after HD LCMV infection, we occasionally detected P14 clusters with some interspersed SMARTA T cells, indicative of interactions on a common DC platform as has been proposed (25, 26).

Our results uncover that alterations in T cell motility parameters precede detectable surface levels of early activation markers, at least during LD infections. The mechanisms underlying changes in dynamic T cell behavior before activation marker expression are not fully understood to date. Its rapidity suggests that it is independent of transcriptional regulation but rather induced by biochemical signals. As example, short Ca-fluxes correlate with transient arrest (35). An open question is whether occasional synapse-like interactions observed in the LD infection model remain a prerequisite for T cell proliferation, or whether transient kinapse-like interactions suffice for activation as has been reported (6). In LD infections, synapses might be rare as a result of less frequent APC. It should be also noted that we did not synchronize dwell times in our experiments by blocking entry of circulating T cells, in order to avoid causing LN hypocellularity during the 72 h observation period. It is therefore likely that our image sequences contain T cells with distinct dwell times, and therefore divergent Ag exposure, within the lymphoid tissue. Nonetheless, at least in the HD LCMV infection model, our flow cytometry analysis suggests most cells became rapidly activated and followed a comparable kinetic throughout the time points analyzed.

We did not address here whether kinapse- and synapse-like behavior correlate with memory T cell formation. Firm T cell arrest appears dispensable for effector T cell generation (6, 36). Yet, absence of ICAM-1 on DCs prevents firm adhesion of CD8^+^ T cells and results in lack of memory formation (37). Similarly, lack of the Cdc42 activator DOCK8 results in defective CD8^+^ T cell memory formation, correlating with defective adhesion to DCs (38). Taken together, there is some evidence that persistent T cell adhesion to DCs may influence memory T cell formation. At the same time, memory precursor effector T cells are thought to experience less inflammatory signals as compared to short-lived effector T cells (39). Thus, the connection between memory formation, DC interaction time, and signals interchanged during prolonged firm adhesion remains incompletely understood.

Taken together, our data show that T cells respond dynamically to varying viral inocula. The dynamic behavior of responding T cells is not hardwired during microbial infections, confirming studies employing peptide-pulsed DC transfer. We consider kinapse- vs. synapse-like behavior of T cells as useful probes to indirectly assess the relative abundance of cognate pMHC levels inside lymphoid tissue during inflammation. Changes in T cell motility parameters emerge as one of the earliest signs of a burgeoning adaptive immune response and reflect the adaptation of T cells to available Ag levels.

## Materials and Methods

### Mice

Ai14 mice, which encode a floxed STOP codon followed by tdTomato reporter gene under control of the ROSA26 promotor (40), were crossed with ZP3-cre mice, which express cre in oocytes (41), to generate “Ai-ZP” mice with ubiquitous red fluorescence in all tissues. SMARTA mice carry a transgenic MHC—II restricted TCR specific for the gp61−80 epitope of the LCMV peptide (42). P14 mice express a transgenic MHC class I restricted TCR specific for the gp33−41 epitope of the LCMV glycoprotein (43). In some experiments, we used “P14-GFP” mice that were derived from crossing the P14 line with Tg 30Scha “Ubi-GFP” mice (44). Male and female 7–9 week-old C57BL7/6 mice were obtained from Janvier (Le Genest-Saint-Isle, France). All animals were maintained in specific pathogen-free conditions at the Department of Biomedical Research, at the Theodor Kocher Institute at the University of Bern and University of Fribourg. All animal work has been approved by the Cantonal Committees for Animal Experimentation and conducted according to federal guidelines.

### Reagents

The fluorescent dyes 5(6)-CFDA, SE (CFSE), (5-(and−6)-(((4-chloromethyl) benzoyl) amino) tetramethylrhodamine) (CMTMR, CellTracker orange) and Chloromethyl-coumarin (CMAC, CellTracker blue) were purchased from ThermoFisher (Basel, Switzerland) and the cell proliferation dye eFluor 670 was from eBioscience (San Diego, CA). mAb against PNAd (MECA-79) was from nanotools (Freiburg, Germany) and coupled to AlexaFluor-633 using a Protein Labeling Kit from Molecular Probes (Basel, Switzerland). Fc receptor blocking mAb (clone 2.4G2, 4.5 mg/mL) in FACS buffer (D-PBS supplemented with 1% milk powder and 0.1% NaN3) was produced in-house. T cells were fixed with 1% paraformaldehyde (PFA; Electron Microscopy Sciences, Lucerne, Switzerland) diluted in D-PBS. D-PBS supplemented with 10 mM EDTA was used for homogenizing LNs for flow cytometry experiments.

### T Cell Purification/Labeling and Adoptive Transfer

Polyclonal T cells were isolated from C57BL/6 or Ai-ZP mice. LNs (popliteal, mandibular, inguinal, brachial, axillary) and spleen were harvested and homogenized using a 70 μm cell strainer. CD8^+^ and CD4^+^ T cells from P14/P14-GFP and SMARTA donors, respectively, were isolated using EasySep negative selection kit (STEMCELL Technologies, Grenoble, France). Polyclonal control T cells were isolated with mouse T cell isolation kit (STEMCELL Technologies, Grenoble, France) according to the manufacturer's protocol. Purity of the isolated T cells was tested using FACSCaliburTM (BD Biosciences) and was typically > 90%. P14 CD8^+^ T cells were labeled with 5 μM CFSE. SMARTA CD4^+^ T cells were labeled with 2.5–5 μM CMTMR and polyclonal T cells from C57BL/6 mice were labeled with 20 μM e670 (for flow cytometry) or 20 μM CMAC (for 2PM imaging) for 20 min at 37°C. For the proliferation assay, P14 CD8^+^ and SMARTA CD4^+^ T cells were labeled with 5 μM CFSE or 2 μM Cell Tracker Violet (CTV). For 2PM intravital imaging, 2–3 × 10^6^ cells of C57BL/6 or Ai-ZP control T cells, P14 CD8^+^ and SMARTA CD4^+^ T cells were transferred i.v. into sex- and aged-matched C57BL/6 recipients 1 day prior to infection.

### Viral Infection

One day after T cell transfer, LCMV Clone 13 was injected s. c. either into the right (for 2PM) or right and left foot hock (for flow cytometry) of C57BL/6 recipient mice at a low (1–10 × 10^2^ plaque forming units, pfu) or high (7 × 10^5^ pfu) inoculum in a volume of 10 μL. The LCMV Clone 13-A3 strain was obtained from the European Virus Archive (https://www.european-virus-archive.com/) and carries a V->A mutation at position 35 of the LCMV gp33-43 peptide, which prevents activation of P14 CD8^+^ cells (21, 22, 45). C57BL/6 recipient mice were co-infected with HD LCMV Clone 13-A3 (7 × 10^5^ pfu) and LD LCMV Clone 13 (1–10 × 10^2^ pfu). For DC activation analysis by flow cytometry, LCMV Clone 13 was injected s. c. in the right (LD), and left (HD) foot hock without previous T cell transfer.

### 2PM of the Popliteal LN

The popliteal LN of recipient mice was surgically exposed for 2PM imaging at 24, 48, and 72 h p.i. as described (32). Immediately prior to imaging, HEVs were labeled by i.v. injection of AlexaFluor-633-conjugated MECA-79. 2PM imaging was performed by using a Trimscope system equipped with an Olympus BX50WI fluorescence microscope and an 20X (NA 0.95, Olympus) or 25X objective (NA 1.10, Nikon) and controlled by the ImSpector software (LaVision Biotec, Bielefeld, Germany). For detection of second harmonic generation of the collagen–rich capsule, a MaiTai Ti: sapphire laser was turned to 840 nm and to 780 nm for excitation of the fluorophores. To perform four-dimensional analysis of cell behavior *in situ*, z-stacks of 11–16 images with 4 μm spacing from areas 200–400 μm^2^ was recorded every 20 s for 20–30 min. Vivofollow software allowed a continuous drift offset correction in real time using fine pattern matching during 2PM imaging (46). Image sequences of z-stacks were converted into volume-rendered four-dimensional videos using Volocity software (Improvision, PerkinElmer, Cambridge, UK). This software was used for semi-automated tracking of cell motility with additional visual check to assure correct tracking. Mean speeds and meandering index, defined as migrated distance divided by euclidian distance between start and end point of the track, were calculated from the (x, y, z) coordinates of the cell centroids. The arrest coefficient was calculated as the percentage per track a cell moved slower than 4 μm/min by using a custom script for Matlab (MathWorks) (32). T cells with the average track speeds below 4 μm/min were considered as synapse-like interacting cells, while cell velocities above 4 μm/min were defined as non-interacting or kinapse-forming T cells.

### Flow Cytometry

At 24, 48, and 72 h p.i., virus-draining popliteal LNs and non-draining brachial LNs were harvested from recipient mice and homogenized using a Corning™ Falcon™ tube with Cell Strainer Snap Cap (5 ml). 2–5 × 10^6^ cells/well were plated on 96-well V-bottom plates and Fc receptors were blocked with 2.4G2 mAb for 20–30 min at 4°C. Cells were stained with fluorochrome—conjugated mAbs CD8 (53–6.7), CD4 (GK1.5), and CD69 (H1.2F3) from BioLegend (San Diego, CA) in 50–100 μl FACS buffer for 20 min at 4°C. Alternatively, cells were stained with biotinylated mAb against CD25 (PC61) followed by secondary staining with fluorochrome-conjugated streptavidin for 20 min at 4°C. For the proliferation assay at 72 and 120 h p.i., cells were stained for with fluorochrome-conjugated mAbs against CD8 (53–6.7) and CD4 (GK1.5). For DC activation analysis at 24 h p.i., cells were stained with fluorochrome—conjugated against mAbs CD11c (N418), MHC class II (M5/114.15.2), CD80 (16–10A1), and CD86 (GL-1) from BioLegend (San Diego, CA) in 30 μl FACS buffer for 30 min at 4°C. Zombie Red™ (BioLegend) was used as viability dye. After staining, the fixation was performed with 1% PFA for 20 min at 4°C. Samples were analyzed using AttuneTM NxT Flow Cytometer and AttuneTM NxT Software (ThermoFisher), FlowJoTM 10 software (Treestar) and GraphPad Prism.

### Statistical Analysis

Flow cytometry data were analyzed using unpaired Mann-Whitney *U*-test or one-way ANOVA (Prism, GraphPad). Significance levels: ^*^ = *p* < 0.05; ^**^ = *p* < 0.01; ^***^ = *p* < 0.001. *P*-values > 0.05 were considered non-significant.

## Data Availability

All datasets generated for this study are included in the manuscript and/or the Supplementary Files.

## Ethics Statement

All animals were maintained in specific pathogen-free conditions at the Department of Biomedical Research, at the Theodor Kocher Institute/University of Bern and University of Fribourg. All animal work has been approved by the Cantonal Committees for Animal Experimentation and conducted according to federal guidelines.

## Author Contributions

SS and XF performed experiments. SS analyzed all experiments and co-wrote the manuscript. JB performed DC activation experiment. NP and DM provided vital material. JS supervised research and wrote the manuscript with input from all coauthors.

### Conflict of Interest Statement

The authors declare that the research was conducted in the absence of any commercial or financial relationships that could be construed as a potential conflict of interest. The reviewer TRM declared a past co-authorship with one of the authors JS.
