# Supplementary material for: Initial Viral Inoculum Determines Kinapse-and Synapse-Like T Cell Motility in Reactive Lymph Nodes
Source: Front Immunol. 2019 Sep 6;10:2086. doi: 10.3389/fimmu.2019.02086 (PMC6743022; doi:10.3389/fimmu.2019.02086)

**Supplementary Information**

**Figure S1. CD4^+^ and CD8^+^ T cell activation and proliferation kinetics in non-draining brachial LNs.** (A) Percentage and MFI of CD69^+^ control (grey) and P14 (green) or SMARTA T cells (blue) post LD LCMV infection. (B) Percentage and MFI of CD25^+^ control (grey) and P14 (green) or SMARTA T cells (blue) post LD LCMV infection. (C) Percentage and MFI of CD69^+^ control (grey) and P14 (green) or SMARTA T cells (blue) post HD LCMV infection. (D) Percentage and MFI of CD25^+^ control (grey) and P14 (green) or SMARTA T cells (blue) post HD LCMV infection. The horizontal red line represents the median. Each dot represents one mice. Data are pooled from three independent experiments with a total of four to six mice per condition and time point. Statistical significance was analyzed using one-way ANOVA test. * = p < 0.05; ** = p < 0.01; *** = p < 0.001.

**Figure S2. DC activation correlates with LCMV inoculum.** DC activation was analyzed by flow cytometry of the respective draining popliteal LNs at day 1 post LD (right foot hock) and HD (left foot hock) LCMV infection. Brachial LNs were analyzed as non-draining lymph nodes (ndLN). (A) Gating strategy for MHC-II^+^ CD11c^+^ DCs. Numbers indicate percentages of gated cells. (B) Flow cytometry histograms of CD80 and CD86 in each condition. (C) Percentage and MFI of CD80^+^ and CD86^+^ MHC-II^+^ CD11c^+^ DCs. Each dot represents data from one mouse. Red lines depict mean. Data in C were analyzed using ANOVA with Tukey’s multiple comparisons test. * = p < 0.05; ** = p < 0.01; *** = p < 0.001.

**Supplemental video legend**

**Video S1.** Representative 2PM video showing control, P14 and SMARTA T cells in non-infected LNs. The nonmotile white cell in the center is likely an autofluorescent macrophage. Scale bar, 20 µm. Time in min:s.

**Video S2.** Representative 2PM video showing control, P14 and SMARTA T cells in LD LCMV-draining LNs at 24 h p.i.. Scale bar, 20 µm. Time in min:s.

**Video S3.** Representative 2PM video showing control, P14 and SMARTA T cells in LD LCMV-draining LNs at 48 h p.i.. Arrowheads depict synapse-like-behavior of P14 (filled) and SMARTA (empty) T cells. Scale bar, 20 µm. Time in min:s.

**Video S4.** Representative 2PM video showing control, P14 and SMARTA T cells in LD LCMV-draining LNs at 72 h p.i.. Filled arrowhead depicts synapse-like-behavior of a P14 T cell. Scale bar, 20 µm. Time in min:s.

**Video S5.** Representative 2PM video showing control, P14 and SMARTA T cells in HD LCMV-draining LNs at 24 h p.i.. Arrowheads depict synapse-like-behavior of P14 (filled) and SMARTA (empty) T cells. Scale bar, 20 µm. Time in min:s.

**Video S6.** Representative 2PM video showing control, P14 and SMARTA T cells in HD LCMV-draining LNs at 48 h p.i.. Arrowheads depict exemplary synapse-like-behavior of P14 (filled) and SMARTA (empty) T cells. Scale bar, 20 µm. Time in min:s.

**Video S7.** Representative 2PM video showing control, P14 and SMARTA T cells in HD LCMV-draining LNs at 72 h p.i.. Arrowheads depict exemplary synapse-like-behavior of P14 (filled) and SMARTA (empty) T cells. The nonmotile white cell (top left) is likely an autofluorescent macrophage. Scale bar, 20 µm. Time in min:s.

**Video S8.** Representative 2PM video showing control, P14 and SMARTA T cells in co-infected HD LCMV Clone 13-A3 and LD LCMV Clone 13-draining LNs at 24 h p.i.. Empty arrowheads depict synapse-like-behavior of SMARTA T cells. Scale bar, 20 µm. Time in min:s.


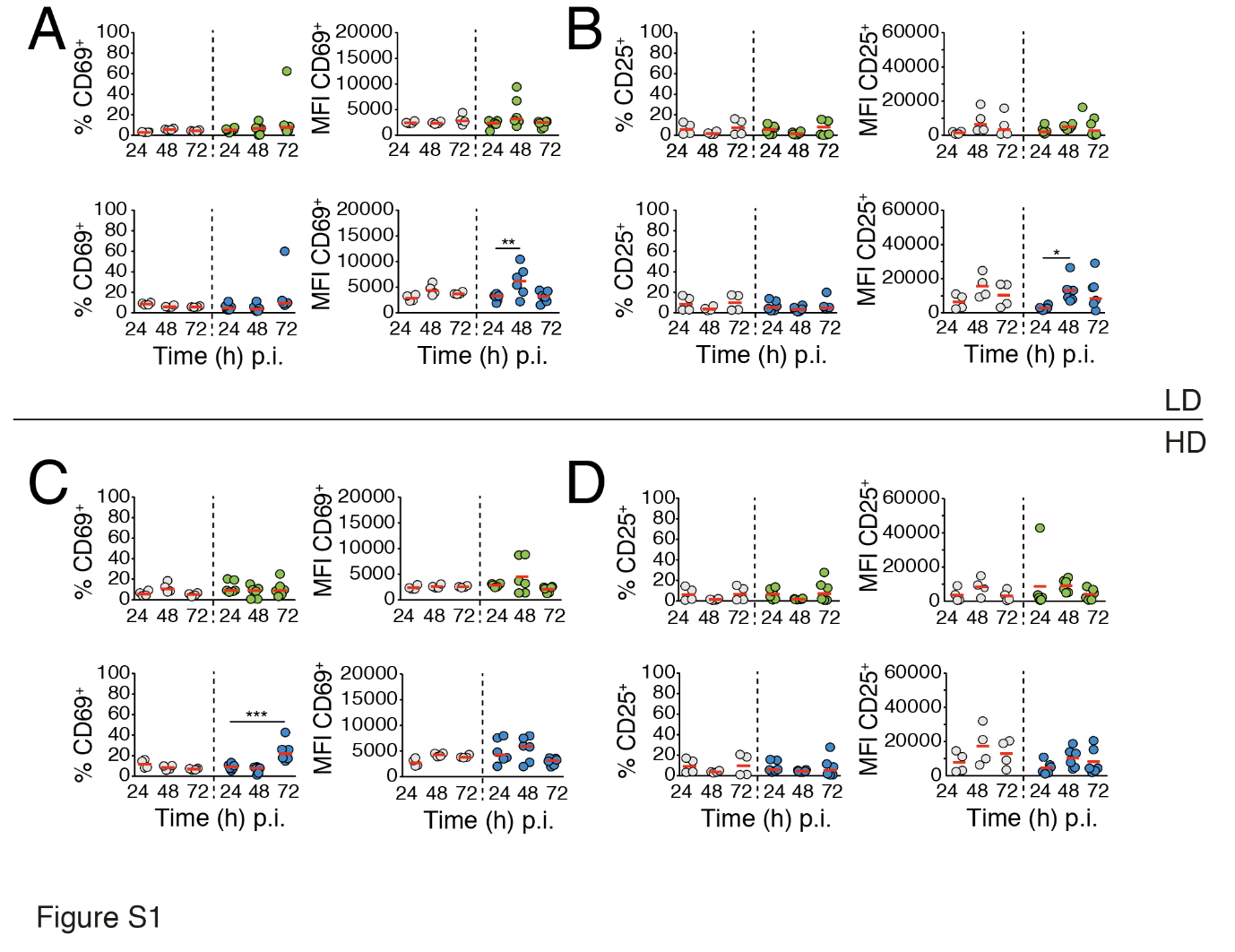


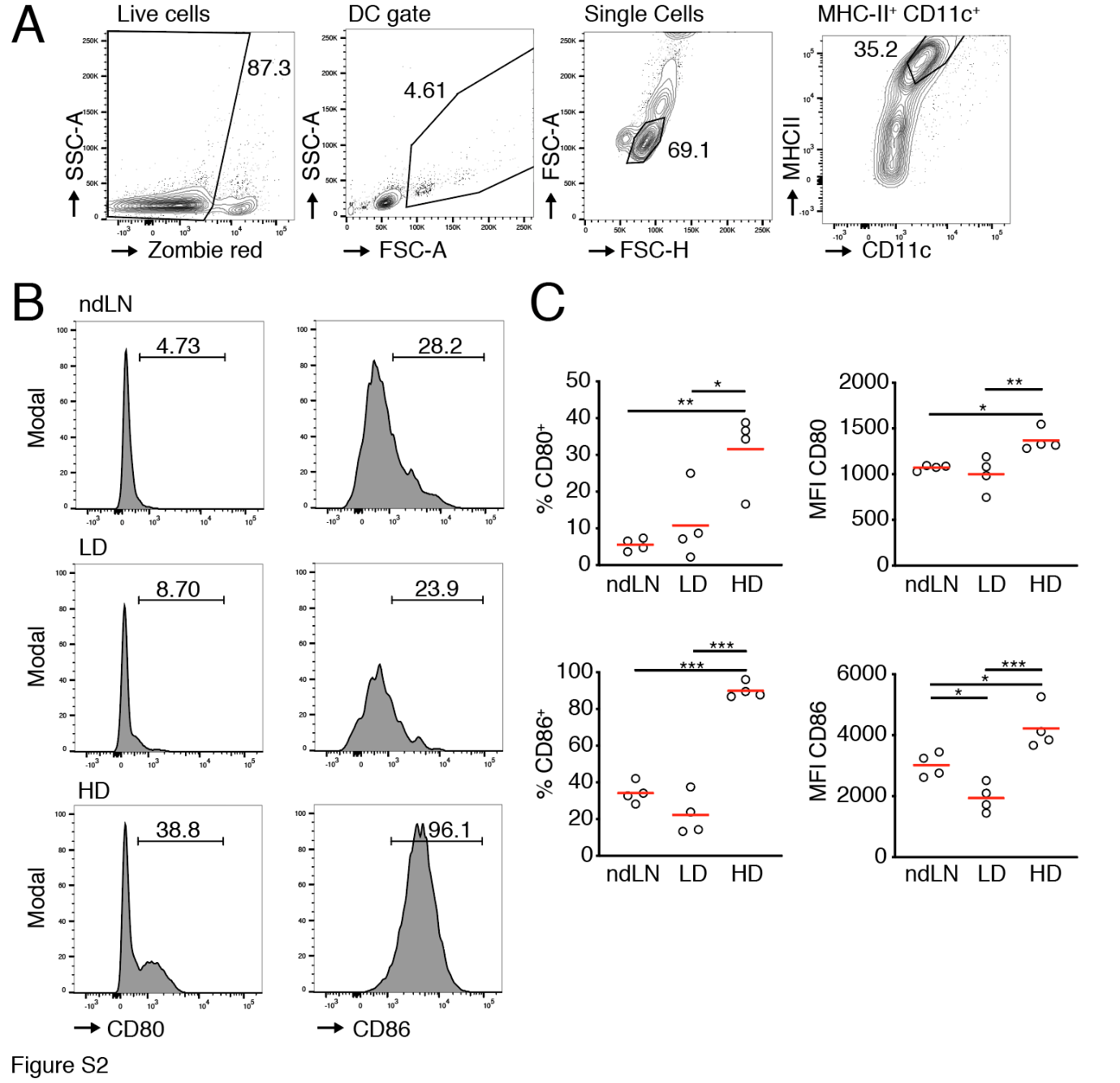

Supplement: Supplementary file 9 [file Data_Sheet_1.docx]
